# No longer uncertain: the validation of tenebrionid insects as hosts of Blattambidensovirus incertum1 isolates by phylogeny and infection studies

**DOI:** 10.1099/jgv.0.002211

**Published:** 2026-02-10

**Authors:** Fang Shiang Lim, Joel González-Cabrera, Johannes A. Jehle, Thomas Lefebvre, Jörg T. Wennmann

**Affiliations:** 1 Julius Kühn Institute (JKI) - Federal Research Centre for Cultivated Plants, Institute for Biological Control, Schwabenheimer Str. 101, 69221 Dossenheim, Germany; 2 Department of Genetics, Institute BIOTECMED, Universitat de València, Dr Moliner 50, 46100, Burjassot, Spain; 3 Ynsect, 1 Rue Pierre Fontaine, 91000 Évry-Courcouronnes, France

**Keywords:** metagenomics, virus taxonomy, long-read sequencing

## Abstract

The mealworm (*Tenebrio molitor*) is one of the most commonly mass-reared insects for food and feed. Monitoring the health status of commercially reared mealworm populations is of great importance for the early detection of entomopathogens and for preventing pathogen outbreaks. Metagenomic screening is a suitable and commonly used method for detecting entomopathogens. The approach used here previously enabled the discovery of the Tenebrio molitor densovirus (TmDV) (family *Parvoviridae*, subfamily *Densovirinae*) in symptomatic larvae. In the present study, the search for TmDV was extended to larvae, pupae and adults of *T. molitor,* including 19 symptomatic and asymptomatic samples obtained from a commercial mealworm mass-rearing facility. The presence of TmDV in all life stages of *T. molitor* was demonstrated, and its relative abundance was quantified using Nanopore sequencing. The infectivity of TmDV to *T. molitor* was demonstrated by isolating viral particles from sample LD2 and feeding them to mealworms. The experiment confirmed *T. molitor* as a susceptible host but showed a rather asymptomatic course of the infection with little effect on larval growth during 56 days of observation. It is hypothesized that this largely covert infection may explain the lack of reports of TmDV in mealworms or other insects, despite its detection in metagenomics surveillance studies of various insectivorous vertebrates. The complete genomes of 15 different TmDV genotypes present in various ratios in the different life stages of *T. molitor* could be reconstructed. Including these genotype sequences in phylogenetic analyses allowed us to re-evaluate the relationship and diversity of previously reported TmDV and related isolates, all belonging to the species *Blattambidensovirus incertum1*. Our findings suggest that *T. molitor* and possibly other insects are susceptible hosts of viruses of *Blattambidensovirus incertum1,* while its occasional detection in metagenomic datasets of insectivorous vertebrates may not represent true densovirus host associations.

**Impact Statement**

Despite the growing importance of insects as an alternative protein source for food and feed, infections of the mealworm *Tenebrio molitor* with Tenebrio molitor densovirus (TmDV) have been detected only recently. In contrast, TmDV sequences were reported in multiple vertebrate metagenomic studies, raising questions about the virus’s host range and its potential impact on vertebrates. By conducting a comprehensive phylogenetic analysis, we show that these insect and vertebrate-associated densoviruses belong to the same species: *Blattambidensovirus incertum1*. Through re-infection experiments, we demonstrate that *T. molitor* is susceptible to TmDV. Hence, we hypothesize that insects are the real hosts of TmDV, while earlier detections of the virus in vertebrates likely reflect viruses from insect prey rather than real infections. Our work clarifies the ecological host range of the virus and helps to resolve the confusion around its host range and classification.

## Data Summary

The data of this study, including the assembled genomes (PV405217 to PV405235 and PV657118 to PV657132), are publicly available at the National Center of Biotechnology InformationSequence Read Archives under the BioProject PRJNA1242519.

## Introduction

Edible insects are a fast-growing market with production volumes expected to reach 500,000 tons by 2030 [1,2]. Larvae of the yellow mealworm *Tenebrio molitor* L. (Coleoptera: Tenebrionidae) are industrially mass-reared to meet the increasing global demand for sustainably produced protein for food and feed. Rearing mealworm larvae is relatively easy, provides an efficient nutrient conversion and has a low environmental impact. Since 2021, the European Food Safety Authority has authorized the use of dried *T. molitor* larvae as novel food for human consumption [3]. Besides *T. molitor*, the most commonly farmed insect species for food and feed include crickets (*Acheta domesticus* and *Gryllus* spp.) and the black soldier fly (*Hermetia illucens*) [4,6].

Rearing of *T. molitor* usually involves large populations at very high densities, which play a critical role in pathogen outbreaks. Such environments may accelerate the spread of microbial infectious agents, causing severe mortality and consequent economic losses [7,8]. Various entomopathogenic bacteria, fungi and viruses have been reported to affect mass-reared *T. molitor* [2,9]. Therefore, approaches for monitoring insect health and detecting pathogens are becoming increasingly important [10]. A particular threat is posed by densoviruses (family *Parvoviridae*, subfamily *Densovirinae*), small non-enveloped, single-stranded DNA viruses causing epidemic outbreaks in insect populations. Densovirus genomes range from 4 to 6 kb and have two major ORFs: one encoding the non-structural (NS) proteins essential for viral replication, and the other encoding the capsid (VP) proteins required for virus assembly [11]. In mass-reared insects, the Acheta domesticus densovirus has been particularly well characterized and has been implicated in the collapse of commercial cricket colonies [12]. Since densoviruses are relatively stable in the environment, spillovers to virus-free insect colonies or even alternate hosts cannot be excluded [12,13].

The first documented case of a natural virus infection in *T. molitor* dates back to 1969, when densovirus-like particles were found in diseased insects using electron microscopy. However, molecular evidence was not reported at that time [14]. Recently, a densovirus known as Tenebrio molitor densovirus (TmDV) was identified in the USA and reported to cause disease symptoms in *T. molitor* populations, specifically in the larval stage [15]. Thereafter, TmDV was reported in a Turkish population of diseased-looking *T. molitor* larvae [16]. In addition to the discovery of TmDV in *T. molitor* larvae, other closely related densoviruses were also recently reported from the superworm (*Zophobas morio*) [16,17]. Prior to these discoveries, another densovirus was detected in a metagenomics study of lung tissue from the Great Tit (*Parus major*) [18]. This virus is now considered the exemplar isolate for the species for *Blattambidensovirus incertum1* [International Committee on Taxonomy of Viruses (ICTV), family *Parvoviridae*, subfamily *Densovirinae*, accessed on 19 May 2025]. Furthermore, multiple densovirus-like viruses were detected in virome studies of various species, including birds [19], bats [20,21] and pangolins [22]. All of these viruses were phylogenetically classified as members of the same species *Blattambidensovirus incertum1*. However, given the limitations of metagenomics approaches and the fact that densoviruses primarily infect invertebrates, it remains uncertain whether their detection in vertebrates reflects genuine virus–host associations, underscoring the relevance of fulfilling Koch’s postulates.

In this study, *T. molitor* larvae, pupae and adults, either symptomatic exhibiting symptoms similar to those recently described for TmDV infections in the USA [15] or asymptomatic, were received from an insect rearing facility in France. Using a Nanopore-based metagenomics approach [10], the presence of TmDV was confirmed in both symptomatic and asymptomatic *T. molitor* samples. The extensive read length of Nanopore sequencing, which encompassed nearly the entire viral genome, allowed the reconstruction and identification of the whole genomes representing multiple TmDV genotypes occurring at different ratios in asymptomatic individuals. A purified preparation of virus particles derived from a single TmDV genotype was subsequently used in re-infection experiments to verify its aetiological role. These experiments demonstrated the ability of TmDV to infect *T. molitor*, highlighting its potential impact on the food and feed insect farming industries, as well as the importance of implementing hygienic measures to manage insect health in insect mass-rearing farms.

## Methods

### Insect samples

**Fig. 1.**
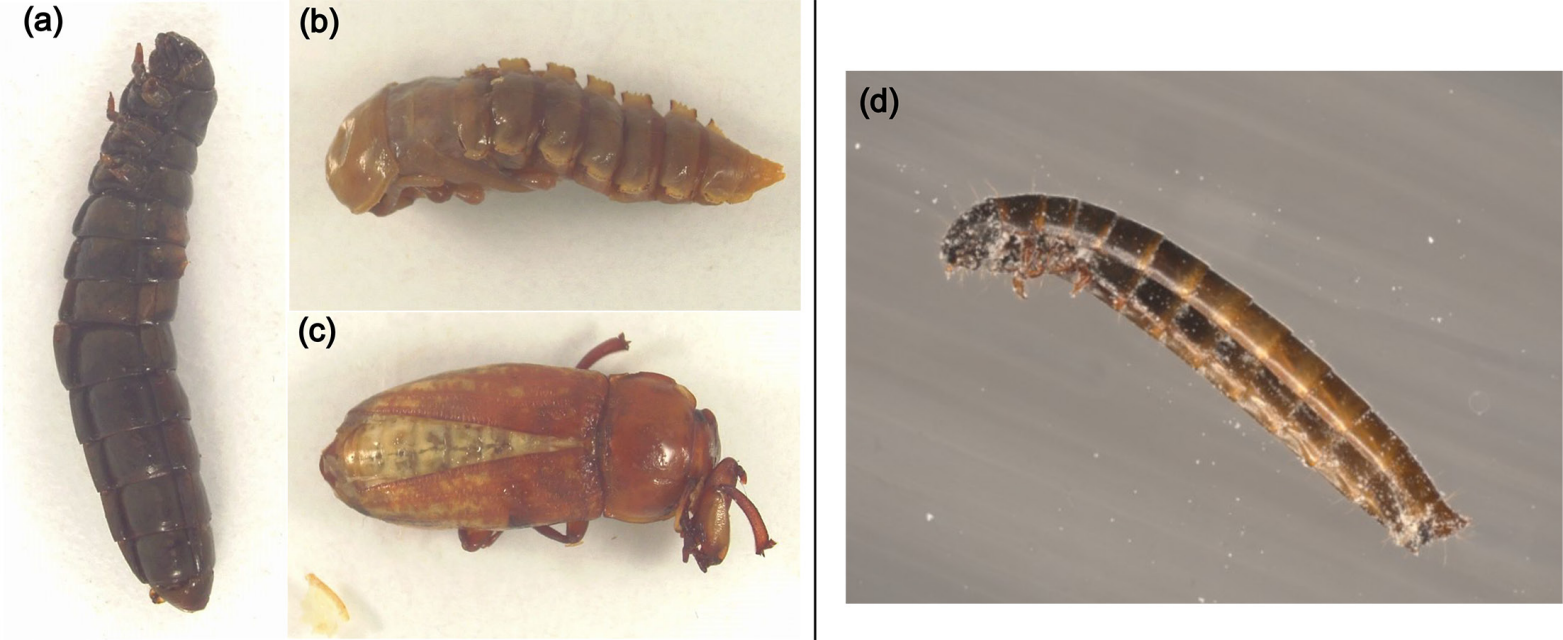
Symptomatic life stages of *T. molitor*: (a) larvae, (b) pupae and (c) adult. Upon visual inspection, the larvae and pupae displayed symptoms of melanization and emitted an unpleasant odour as a sign of degradation. (d) A larva shortly before death from the bioassay experiment with TmDV-LD2 conducted in this study. The larva showed signs of melanization in the head region.

Fifteen symptomatic *T. molitor* samples, five of each life stage, were provided by a commercial mass-rearing facility from France, which reared mealworm for food and feed. Additionally, asymptomatic samples from each life stage were received to serve as controls. All individuals were received dead and were stored at −20 °C until further use. The following sample designations reflecting the status of the individuals were used: L, larvae; P*,* pupae; A, adult; D, symptomatic (apparently diseased) (Fig. 1a-c); and H, asymptomatic (apparently healthy), followed by sample number, e.g. LD1 indicates sample 1 of a dead larva.

### Total nucleic acid extraction

Individual larvae, pupae and adults of *T. molitor* were excised transversally into halves. The anterior part of each sample was subjected to total nucleic acid (DNA and RNA) extraction (ZymoBIOMICS DNA/RNA Miniprep Kit (R2002), Zymo Research Corp., Irvine, CA, USA) following the manufacturer’s protocol with the following modifications. Prior to homogenization, each insect sample was surface-sterilized with 0.5% (v/v) sodium hypochlorite solution for 30 s and subsequently washed in 70% ethanol for 1 min and distilled water for 2 min to remove surface contaminants. The insect sample was then transferred to a microcentrifuge tube containing ceramic beads (0.1 and 0.5 mm) and homogenized on an MP Fastprep-24^™^ (MP Biomedicals, USA) at 6.5 ms^−1^ for 30 s. The tube was rested on ice for 1 min and homogenized again at 6.5 ms^−1^ for 30 s. Prolonged homogenization was avoided to minimize DNA fragmentation. The DNA was eluted in 75 µl of ZymoBIOMICS^™^ DNase/RNase-free water. The quality and quantity of the extracted DNA were assessed using gel electrophoresis (1% agarose; TAE buffer system) and a Quantus^™^ fluorometer (Promega, Wisconsin, WI, USA) using Quanti Fluor^®^ ONE dsDNA system, respectively. The extracted nucleic acids were stored at −80 °C until further use.

### Metagenomics analysis

To screen for entomopathogens, the extracted DNA was prepared for metagenomics analysis using Nanopore sequencing following the method described by Lim *et al*. [10]. DNA library preparation was achieved by using the Ligation Sequencing Kit (SQK-LSK109) coupled with the Native Barcoding Expansion 1–12 kit (EXP-NBD104) for multiplexing of samples, according to the manufacturer’s protocol (Oxford Nanopore Technologies, Oxford, UK). All clean-up steps during library preparation were conducted using Agencourt AMPure XP beads (Beckman Coulter, Indianapolis, IN, USA). The DNA libraries were loaded onto FLO-MIN106D (R9.4.1) flow cells on a MinION Mk1b device (Oxford Nanopore Technologies, Oxford, UK). Basecalling was performed using the high-accuracy model using Guppy v4.5.3 (https://github.com/topics/guppy) on a NVIDIA Jetson Xavier AGX Developer Kit (NVIDIA, Santa Clara, CA, USA). First, reads were filtered with a Phred quality score >7 and a minimum length of 100 nt.

To remove host sequence information, the quality-filtered reads were mapped against the genome of *T. molitor* (NCBI RefSeq assembly GCF_963966145.1) [23]. Unmapped reads were extracted and taxonomically assigned using Kraken2 v2.1.1 [24] against an in-house custom-built index (RFGINV_JKI_ID 1.0) [10]. This index consisted of reference nucleotide sequences from the domains Bacteria and Archaea, the kingdom Fungi, and the superkingdom Viruses (https://ftp.ncbi.nlm.nih.gov/genomes/refseq/) and was retrieved from the National Center for Biotechnology Information Reference Sequence Database (NCBI RefSeq) (created on 27 May 2023). The minimum confidence score threshold for taxonomic assignment was set to 0.05 to remove false positive assignments. Kraken2 output was imported into the Pavian interactive web application (https://github.com/fbreitwieser/pavian) [25] to visualize the respective taxa present in each sample.

### TmDV genome assembly

Sequence reads that were assigned to TmDV [NCBI taxonomy entry name: Parus major densovirus (PmDV); NCBI taxonomy ID: 1907771; naming according to ICTV: *Blattambidensovirus incertum1* (accessed on 3 September 2024)] were extracted from the Kraken2 output using KrakenTools v1.2 (https://github.com/jenniferlu717/KrakenTools; extract_kraken_reads.py script) [26]. For each sequenced sample, a *de novo* genome assembly was performed using the extracted reads. Metagenome-assembled genomes (MAGs) of TmDV were generated from the MinION reads using Canu v2.2 [27] with default parameters for ONT sequencing (-nanopore-raw), with an estimated genome size of 6 kb. Extracted reads were then mapped back to the draft genome using minimap2 v2.17 with the map-ont parameter and were sorted using Samtools v1.9 [28]. Coverage of the assembly was calculated using Qualimap v2.2.1 [29].

### Phylogenetic analysis

For phylogenetic analysis, the *de novo* assembled TmDV genomes were aligned using MAFFT v7 webserver (https://mafft.cbrc.jp/alignment/server/) [30] together with other *Densovirinae* sequences publicly available at NCBI GenBank. The multiple sequence alignment was subjected to gblock trimming to remove poorly aligned regions and to preserve conserved regions. The alignment was then converted to Nexus format, and the best nucleotide substitution model was calculated using ModelFinder, as part of the submodule of IQ-TREE [31]. Bayesian inference trees were then constructed using BEAST [32] with TN+F+I+G4 as the best-fit model according to the Bayesian information criterion.

### Intra-sample variation and genotyping of TmDV

For each sample, the intra-sample variation of TmDV was assessed by aligning the extracted TmDV reads from each sample to the PmDV reference genome (GenBank accession: KU727766.1) using minimap2 (version 2.28, map-ont preset) [33]. Variant calling was performed using Clair3 (version 0.1.12) [34] to identify single-nucleotide variants (SNVs). The resulting VCF files were normalized using bcftools norm (version 1.9) [35], which left-aligns indels to ensure standardized variant representation. Only variant positions with a QUAL score larger than 2 were retained using SnpSift (version 4.3) [36] for downstream analysis. The occurrence of nucleotide frequencies in these variant positions was recorded using mpileup (version 1.16.1) [28] and visualized in R (R v 4.2.1 in RStudio v2022.07.1+554).

These SNV positions then served as anchors to partition TmDV reads from each sample via single linkage analysis. In brief, for each sample, a matrix of reads by SNV positions was constructed and used to compute pairwise dissimilarity scores (D): *D* < 0.5 indicates matching overlaps, *D* > 0.5 indicates mismatches and *D*=0.5 denotes no overlap. Hierarchical clustering with single linkage clustering was performed in R, and continuous clades were extracted for *de novo* assembly using Canu v2.2 [10]. For this analysis, only reads between 2,500 and 5,500 bp in length (~50 % to nearly the full length of the genome) and a maximum of 1,000 TmDV reads were used to ensure that genotypes could be reliably constructed from a minimal number of reads from a pool of TmDV with different genotypes in each sample, if present.

### Purification and re-infection of TmDV

As described above, the *T. molitor* individuals were divided into two halves. Whereas the anterior half was used for nucleic acid extraction (see section above), the posterior half of the symptomatic larva LD2 was used to purify TmDV and perform re-infection experiments. Larva LD2 was transferred into a sterile screw cap tube filled with 800 µl of DNase- and RNase-free water and containing one 4 mm and three 2 mm glass beads. The sample was homogenized using an MP Fastprep-24^™^ (MP Biomedicals, USA) at 6.5 ms^−1^ for 1 min. Subsequently, the homogenate was filtered first through a 0.45 µm and then a 0.2-µm Whatman^™^ Puradisc^™^ FP 30 CA-S syringe filter to remove non-viral particles. The filtered virus suspension was transferred to a Quick-Seal^™^ centrifuge tube (Beckman) and ultracentrifuged at 285,000 rcf (44,333 r.p.m.) for 2 h in a fixed-angle rotor (type 70.1 Ti) on an LB-70M ultracentrifuge (Beckman). After centrifugation, the supernatant was discarded, and the pellet was resuspended in 100 µl nuclease-free water. The quantity of TmDV was measured using the quantitative PCR (qPCR) assay described below.

After quantification, the TmDV stock suspension was diluted from 4.56×10^9^ to 4.56×10^5^ total virus particles using a 10-fold dilution series. From each dilution step, 10 µl virus suspension was mixed with ~5 mm³ of *T. molitor* artificial diet (wet wheat bran). Fifteen larvae weighing between 10 and 60 mg were starved for 24 h prior to being fed the virus-containing diet. Then, the larvae were kept on a virus-free diet for 56 days at 25 °C and 12:12 h light/dark. The infection experiment included a negative control of 15 larvae that were fed a virus-free diet. Every 3 days, the weight and vital status of the larvae were recorded, and the weight gain was calculated with the following formula:


$$Weightgain\left(\%\right)=\frac{Currentweight-Startingweight}{Startingweight}\times 100$$

After 56 days, the experiment was stopped, and all larvae were subjected to DNA extraction using Ron’s Tissue and Blood DNA Mini Kit (Römerberg, Germany) following the manufacturer’s protocol. The extracted DNA was subjected to PCR using ORF5 (NS3)-specific oligonucleotide primers (Table 1) to examine the presence of the virus in the insects after re-infection.

**Table 1.** Pairs of oligonucleotides used to amplify fragments of the five TmDV ORFs. The positions indicate the locations of the oligonucleotides in relation to the TmDV genome (NCBI GenBank accession no MW628494). For NS1, an additional primer was designed for qPCR analysis

Target	Name	Position	Amplicon size (bp)	Sequence (5′-3′)
ORF5 (NS3)	prTmDV_ns3_fw	300–324	691	AGACTATCCAGAGGGAACTACTACT
	prTmDV_ns3_rv	995–990		GCATCTCCCAGAGTACTGTCAAAATA
ORF4 (NS2)	prTmDV_ns2_fw	845–867	938	CATACTGTTATGCAAGGGATTCG
	prTmDV_ns2_rv	1759–1782		GATAATGGACAAACAGCATACCTC
ORF3 (NS1)	prTmDV_ns1_fw	1614–1635	974	CGCTTGACAATAACAATGAACG
	prTmDV_ns1_rv	2566–2587		CCTAATGTATATGGCAGGATGC
ORF1 (VP1.1)	prTmDV_vp1.1_fw	2488–2510	939	GCCACGACCTGATTCTGTAATGA
	prTmDV_vp1.1_rv	3403–3426		CATATTCTGATACTGAACCCATGC
ORF1 (VP1.2)	prTmDV_vp1.2_fw	3350–3372	1,016	CTTGAGACCATCTCGATATTGGT
	prTmDV_vp1.2_rv	4344–4365		CACGCAGTCGAACATTTAAGCG
ORF2 (VP2)	prTmDV_vp2_fw	4222–4246	1,001	GGTTCCATTGTTCCATAGCATAACG
	prTmDV_vp2_rv	5199–5222		GCTTATGGGCAAGCACCAAGTAAG
ORF3 (NS1) qPCR	prTmDV_ns1_qpcr_fw	1015–1037	89	CGGGAGTATGGTGGAAGATTATG
	prTmDV_ns1_qpcr_rv	1084–1103		CTTGCTCCAACGCCCTATTA

### PCR-based densovirus detection

### qPCR assay

For the quantitative detection of TmDV, a primer pair was specifically designed to amplify an 89 bp fragment from ORF3 (NS1) (Table 1), which was cloned into vector pEX-A128. The vector and cloning were ordered from Eurofins Genomics GmbH (Ebersberg, Germany). The pEX-A128::TmDV-NS1 (89 bp) construct was provided at a concentration of 50 ng µl^−1^. The mass of a single DNA construct was calculated using the following formula:


$$mass\;(g\;molecule^{-1})=\left(Length\;of\;Fragment\;(2061\;bp)\right)\times \frac{1\;mol}{6.022\times 10^{23}}\times 660\;g\;{mol}^{-1}\;{bp}^{-1}$$

A serial dilution of the plasmid was created in triplicate, ranging from 10^10^ to 10^2^ copies, to generate a standard curve for the PCR-based quantification of absolute genome copies (=virus particles). This standard curve was used for two different steps of quantification: (i) determining the number of NS1 transcripts in the extracted RNA of all individuals and (ii) estimating the number of virus particles of individual LD2 based on the extracted DNA.

For NS1 transcript determination, 1 µg RNA from each sample was transcribed into cDNA using oligo (dT) primers and random hexamers, following the manufacturer’s protocol in the Prime-Script RT Reagent Kit (Perfect Real Time from Takara Bio Inc, Otsu, Shiga, Japan). This step included a DNase treatment to digest any genomic dsDNA and TmDV ssDNA.

The reaction volume for cDNA synthesis was 20 µl, of which 1 µl was used for each qPCR. In a second quantitative analysis, the above-generated standard was used to estimate the number of virus particles from the purified TmDV from sample LD2. Here, 1 µl of purified virus was directly used as a DNA template. The quantity of TmDV in sample LD2 was directly derived from the standard curve.

All qPCRs were performed in a final volume of 25 µl containing 1× Maxima SYBR Green/ROX qPCR Master Mix and 0.3 µM of forward and reverse primer. The qPCRs were run on a Bio-Rad CFX98 system. The cycling conditions initiated with a denaturation step at 95 °C for 10 min, followed by 35 cycles of denaturation at 95 °C for 15 s, annealing at 48 °C for 30 s and elongation at 72 °C for 30 s.

### Transmission electron microscopy image of TmDV

Drops of the purified TmDV LD2 suspension were applied onto a collodion-covered electron microscope grid using a sterile Pasteur pipette. After 10 s, excessive liquid was removed using filter paper. Then, a drop of 1% aqueous phosphotungstic acid (pH 7.2) was applied onto the grid. After drying, the sample was observed under a JEOL JEM1400 transmission electron microscope at the Electron Microscopy Core Facility at the University of Heidelberg to look for densovirus-like particles.

## Results

### Metagenomic analyses of *T. molitor*

**Fig. 2.**
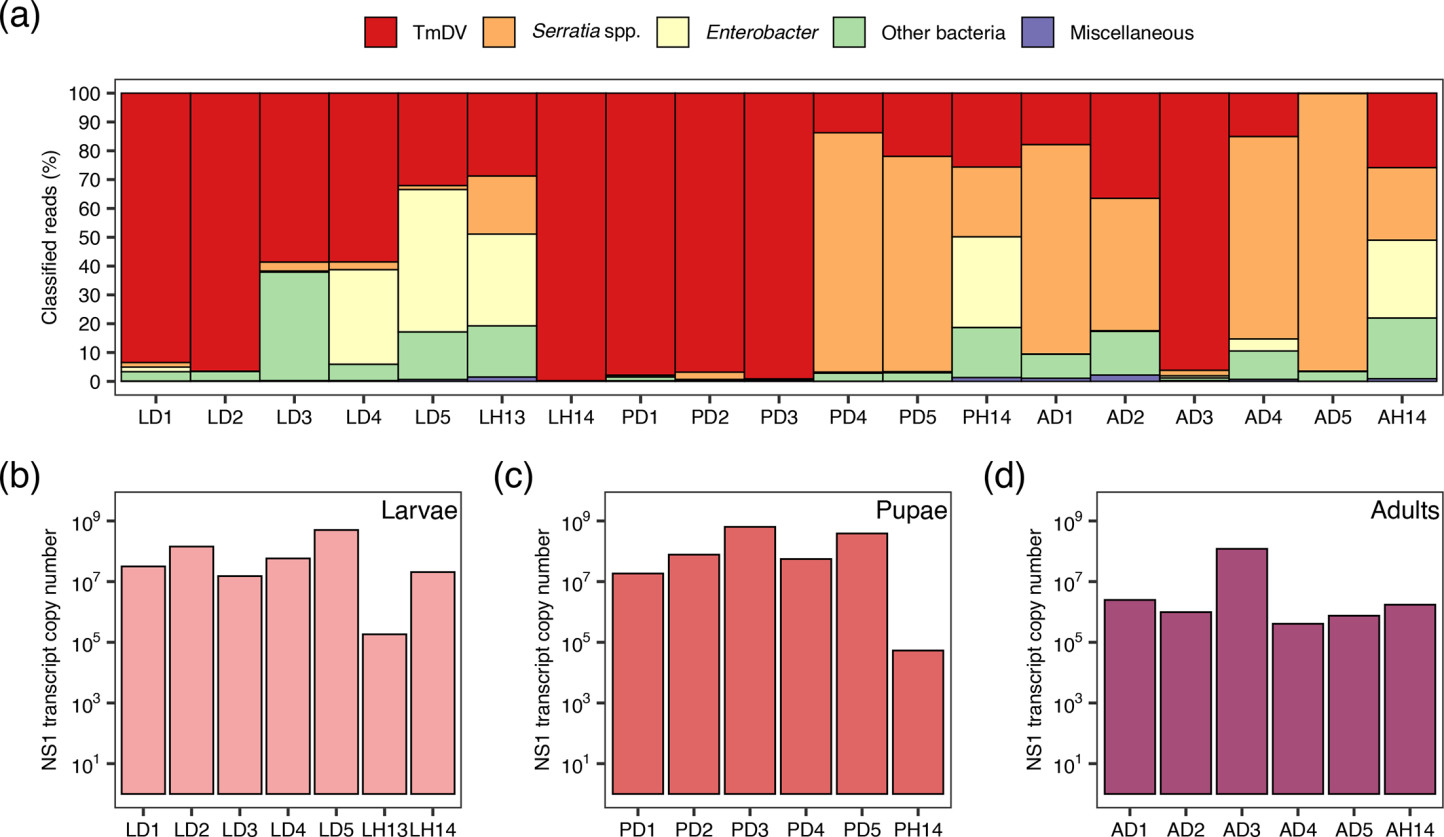
(a) Metagenomics detection and relative abundance of TmDV, as well as associated microbial taxa in larvae (L), pupae (P) and adults of *T. molitor* based on data from Nanopore sequencing. Samples are further categorized as symptomatic (D) or asymptomatic (H). Taxa included *Serratia* spp., *Enterobacter*, other bacteria and miscellaneous groups. (b–d) Quantification of TmDV NS1 transcript copy number of (b) larvae, (c) pupae and (d) adults using reverse transcriptase qPCR. Transcript abundance is plotted on a logarithmic scale, highlighting variable viral load across developmental stages and health status.

Fifteen symptomatic and four asymptomatic *T. molitor* samples (larvae, pupae and adults) were selected from a mass-rearing facility and analysed for the presence of potential entomopathogens, such as TmDV, *Serratia* spp. and *Enterobacter*, by pursuing a Nanopore-sequencing-based metagenomics analysis (Table 2, Fig. 2a). Before the reads were assigned to taxa, the host genome-specific information, which made up between 31% and 98% of all sequencing reads, was extracted and removed from the data. Reads that were not assigned to the host were classified metagenomically, which led to the detection of TmDV in various samples. The proportion of TmDV reads compared to the total number of reads per sample ranged from <0.1% (most adult diseased samples=AD) to >20% (larval diseased samples=LD1, LD2 and LD5) (Table 2). A smaller overall proportion of TmDV reads was found in adult individuals (AD1–AD5) compared to symptomatic larvae (LD1–LD5) and pupae (P1D–PD5), suggesting a lower virus load in the adult life stage. Furthermore, except for asymptomatic LH14, the proportion of reads was higher in the symptomatic than in the asymptomatic individuals. For each of the 19 samples, the TmDV consensus sequences were assembled and uploaded to NCBI GenBank (Table 2).

**Table 2.** Read statistics on the nanopore sequenced samples of *T. molitor*. The percentages refer to the total number of sequenced reads. Reads that were assigned taxonomically to TmDV were extracted and *de novo* assembled to obtain individual TmDV consensus genomes. The TmDV consensus genome sequences were annotated and uploaded to NCBI GenBank

Sample	Total no. read	N50	NCBI SRA accession no.	Reads mapped to *T. molitor*		Reads assigned to TmDV		NCBI GenBank accession no.
LD1	2,196,928	472	SRR32875732	1,556,822	70%	495,590	23%	PV405223
LD2	2,535,030	5375	SRR32875731	1,808,436	71%	488,809	20%	PV405224
LD3	231,647	768	SRR32875721	169,330	73%	22,368	10%	PV405225
LD4	256,565	519	SRR32875720	159,536	62%	43,800	17%	PV405226
LD5	2,104,969	496	SRR32875719	644,997	31%	423,381	20%	PV405227
LH13	390,425	852	SRR32875718	376,703	96%	462	0.12%	PV405228
LH14	1,899,016	413	SRR32875717	1,577,315	83%	251,241	13%	PV405229
PD1	834,367	350	SRR32875716	777,798	93%	19,210	2.3%	PV405230
PD2	2,050,490	314	SRR32875715	1,693,207	82%	212,455	10%	PV405231
PD3	254,579	417	SRR32875714	208,057	82%	29,891	12%	PV405232
PD4	788,244	5418	SRR32875730	102,315	13%	89,147	11%	PV405233
PD5	746,072	5407	SRR32875729	66,829	9%	127,422	17%	PV405234
PH14	347,550	1235	SRR32875728	339,157	98%	214	<0.1%	PV405235
AD1	914,413	508	SRR32875727	881,497	96%	602	<0.1%	PV405217
AD2	2,450,874	464	SRR32875726	2,371,224	97%	982	<0.1%	PV405218
AD3	225,556	416	SRR32875725	204,908	91%	6103	2.7%	PV405219
AD4	249,159	477	SRR32875724	237,999	96%	115	<0.1%	PV405220
AD5	754,328	2049	SRR32875723	132,543	18%	1013	0.13%	PV405221
AH14	489,598	685	SRR32875722	473,943	97%	311	<0.1%	PV405222

In addition to TmDV, further reads were assigned to the genera *Serratia*, *Enterobacter* and other micro-organisms (Fig. 2a). The majority of the *Serratia* reads were assigned to *Serratia marcescens* at the species level. Among the classified reads, no other taxa that might be associated with potential entomopathogens were found. Besides DNA, the RNA was also extracted from the individual *T. molitor*. The RNA was used to detect transcripts that provided evidence of replication activity of TmDV within the host. A fragment of ORF3 (NS1) was selected and detected by qPCR in symptomatic and asymptomatic larvae, pupae and adults (Fig. 2b - d). The number of NS1 transcripts varied between 10^6^ and 10^10^ copies, indicating that transcripts for the non-structural protein NS1 were actively transcribed. This demonstrated that the TmDV genes required for replication are actively transcribed across all life stages of the mealworm.

### Isolation and re-infection of TmDV

**Fig. 3.**
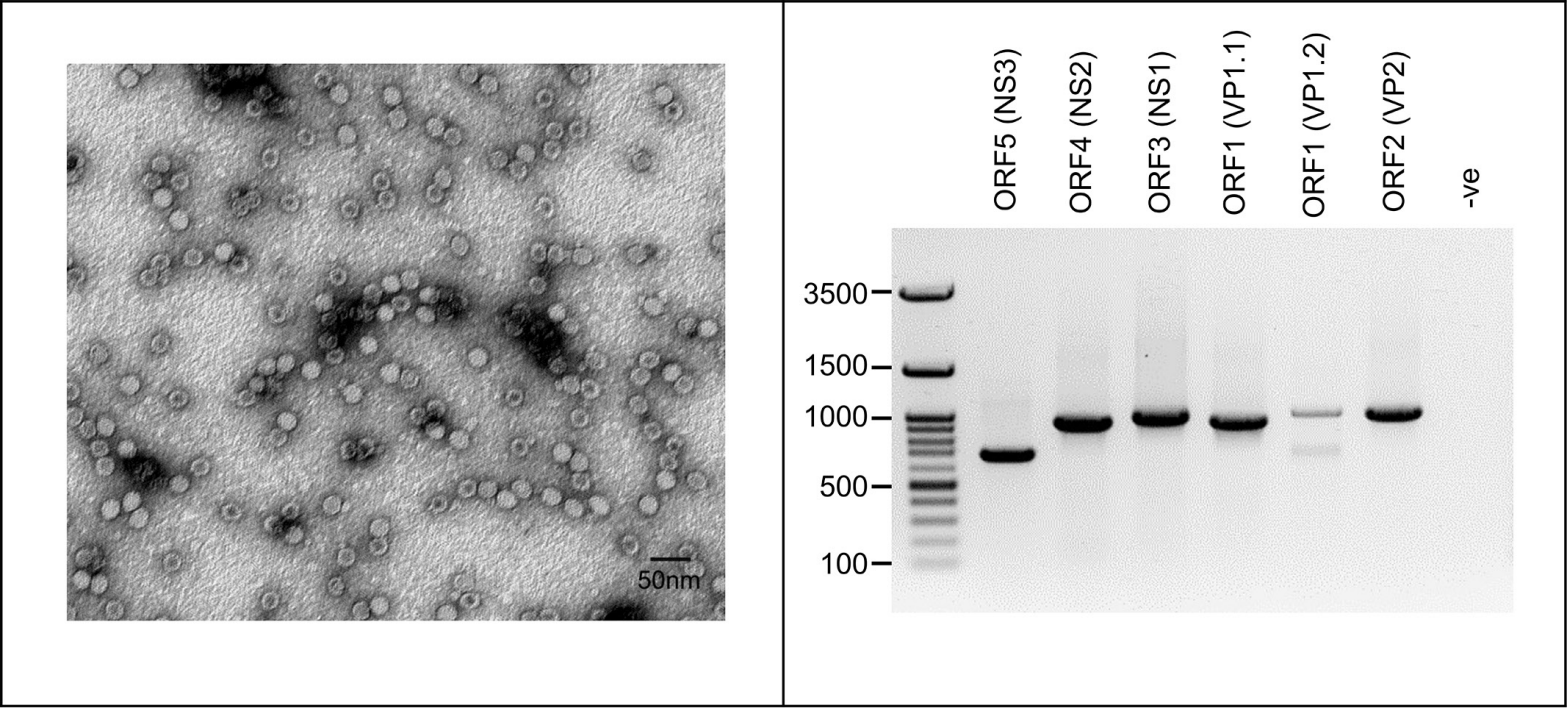
Electron microscopy image of purified TmDV that was isolated from the *T. molitor* sample LD2 (left). PCR-based amplification of TmDV-LD2 fragments with six primer pairs (Table 1) targeting all five ORFs (right). Lane -ve indicates negative control.

Since most larval tissue was available for *T. molitor* LD2, this sample was chosen for TmDV purification using ultracentrifugation. The presence of TmDV-LD2 particles and the purity of the preparation were assessed by transmission electron microscopy, showing small, icosahedral, non-enveloped virus particles of ~25 nm in diameter (Fig. 3, left). To confirm the identity of TmDV-LD2, a PCR was designed with six pairs of primers targeting the five ORFs of TmDV (Table 1). The primers were designed to ensure that the amplified fragments not only comprise each TmDV ORF but also overlap with one another, allowing rapid amplification, as well as sequencing and reconstructing the full TmDV genome. The location of the PCR primers omitted the terminal repeat regions of the TmGV genome but covered about 50 to 100 bp of the non-coding regions upstream and downstream of both terminal ORFs. This PCR and Sanger sequencing-based approach facilitated the detection of TmDV based on its whole genome and established a diagnostic tool for efficiently and quickly monitoring the abundance of the virus in mealworm rearing facilities. In addition, the PCR primer sequences were optimized to work at a common annealing temperature of 50 °C, resulting in a single, specific amplification product for each primer pair. Only for ORF1 (VP1.2), a second, non-specific band was observed (Fig. 3, right). After purification of the specific PCR fragments from the gel and Sanger sequencing them, the entire TmDV-LD2 genome sequence was assembled and confirmed with the reads from the metagenomics study of the LD2 sample (NCBI accession no. PV405224.1).

**Fig. 4.**
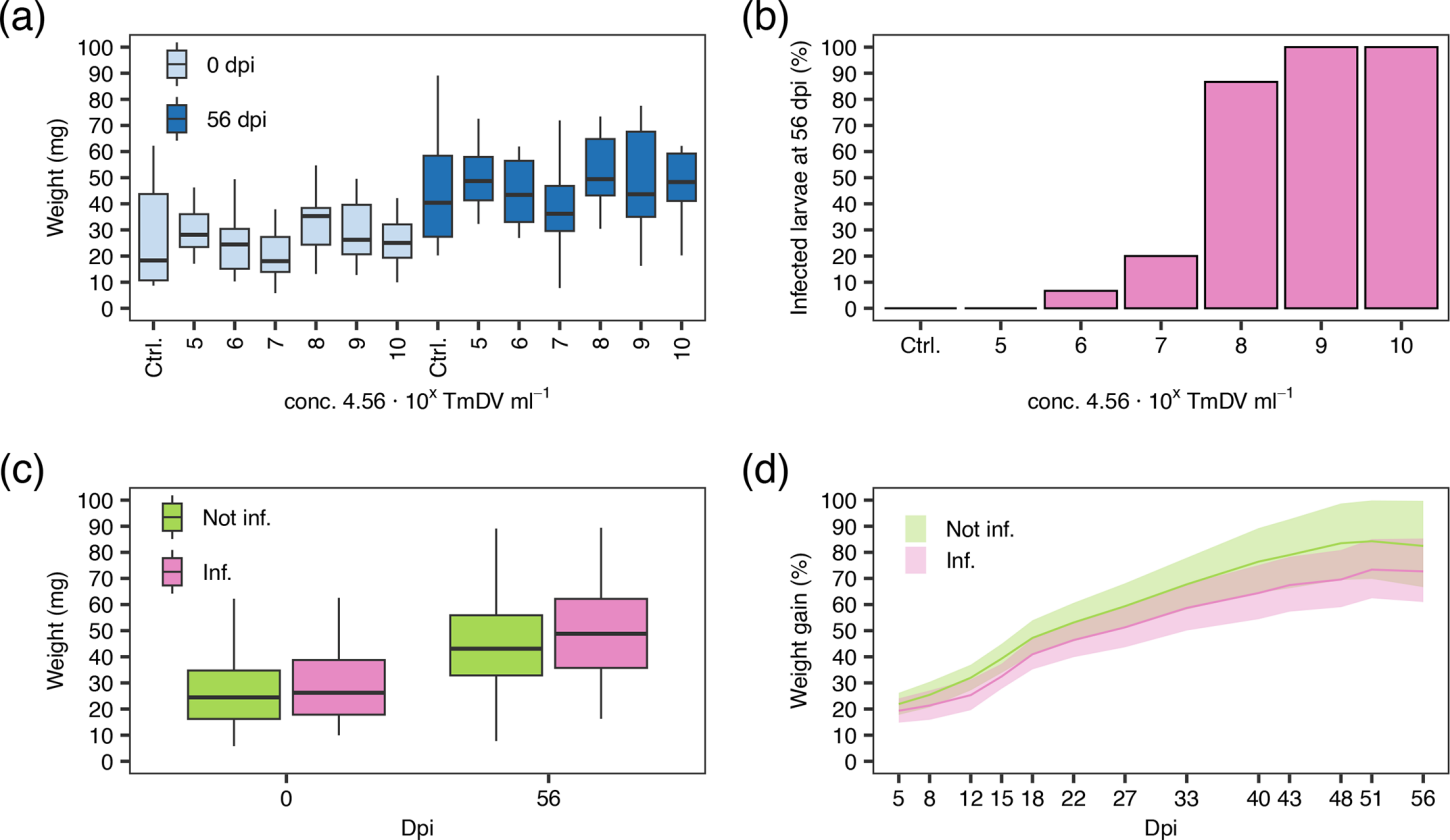
(a) Weight and weight gain/loss analysis of *T. molitor* larvae treated with different doses of TmDV-LD2. The weight of the larvae in the individual treatments at 0 and 56 days post infection (dpi). As the larval rearing was not synchronized, the initial weight at 0 dpi was used as a reference for all analyses. (b) Percentage of infected larvae in the bioassay treatments. TmDV-LD2 was detected by PCR analysis. (c) Weight of larvae separated according to their infection status. (d) Weight gain of infected and uninfected mealworms. Weight gain was calculated for each day relative to the initial weight (0 dpi). The geometric mean with 95% confidence intervals is shown.

In the next step, the infectivity of TmDV-LD2 to *T. molitor* larvae was assessed. First, the concentration of TmDV-LD2 was estimated using a qPCR assay based on plasmid pEX-A128::TmDV-NS1 (89 bp), which contained a single copy of an 89 bp fragment of the ORF3 (NS1) as a standard. Since each densovirus virion contains a single genome copy, it was assumed that the number of measured DNA copies equals the number of virions in the sample. Based on this assumption, the TmDV-LD2 concentration was estimated to be 4.56×10^9^ virus particles (). Then, five different doses ranging from 4.56×10^10^ to 4.56×10^6^ TmDV-LD2 particles were fed to *T. molitor* larvae, resulting in only two dead larvae by day 13 and day 18 at the highest concentration within the entire bioassay experiment that lasted 56 days. Prior to death, the two moribund mealworms showed sluggish movement, slow growth, signs of melanization and reduced weight gain compared to uninfected individuals, proving *T. molitor* as a susceptible host and thereby confirming Koch’s third postulate (Fig. 1d). Besides mortality, the weight of the TmDV-LD2 treated larvae and uninfected controls was measured regularly to assess a potential effect of the virus on the growth of the host (Fig. 4a). Because the virus-free mealworm rearing was not synchronized, the larvae differed in their developmental stages at the start of the experiment, small larvae were selected and their weight was used to approximate the larval status. The starting weight (0 dpi) of all larvae ranged between 10 and 60 mg. It was then compared to larval weight of the different treatment groups, which was not normally distributed in all treatments (Shapiro–Wilk test, *P*<0.05). It was assumed that the initial weight was the same for all larvae in all treatments, as there were no differences in weight at the start of the bioassay (Kruskal–Wallis test, *χ*² = 7.68, df = 6, *P*=0.263) (Fig. 4a). The larval weight at 56 dpi ranged between 30 and 90 mg but also did not differ between treatments (Kruskal–Wallis test, *χ*² = 7.27, df = 6, *P* = 0.288). Testing for weight differences within each treatment revealed a significant increase of weight between 0 and 56 dpi (Wilcoxon rank-sum test, *P* < 0.05) (Fig. 4a). All treatments resulted in the same larval weight at the end of the bioassay, regardless of the amount of TmDV applied as inoculum (Fig. 4a). On the other hand, the PCR analyses of all inoculated larvae using the primers prTmDV_ns3_fw and prTmDV_ns3_rv for ORF5 (NS3) (Table 1) revealed that at day 56, none of the individuals in the control group or in the treatment with the lowest TmDV dose was virus-positive. With increasing TmDV dose, the number of PCR-positive infected individuals rose from 6.7% (4.56×10^6^ TmDV particles), 20% (4.56×10^7^ particles) and 86.7% (4.56×10^8^ particles) to 100% in the treatments with the two highest doses (Fig. 4b). However, only at the highest dose of 4.56×10^10^ TmDV-LD2 particles two individuals show typical infection symptoms and died. The detection of uninfected and infected *T. molitor* larvae allowed analysing the weight of the larvae separately, regardless of their infection status (Fig. 4c), showing that the larvae at 0 dpi (Kruskal–Wallis test, *χ*² = 1.52, df = 2, *P* = 0.218) but also at 56 dpi (Kruskal–Wallis test, *χ*² = 2.1, df = 2, *P* = 0.147) did not differ in weight. Thus, 56 days after the start of the experiments, the infected *T. molitor* were not heavier or lighter than uninfected control individuals. Finally, when the weight gain and growth dynamics of infected individuals and the untreated control group were analysed over the duration of the experiment (Fig. 4d), it was found that the uninfected larvae tended to have a slightly higher percentage weight gain than infected ones, but the interaction between time (Day) and treatment was not significant in the linear model (ANOV: Day × Treatment, *F* (6, 1332) = 0.31, *P* = 0.93). After 56 dpi, the weight increase was 82.5% (95% CI 66.7–99.7%) and 72.7% (95% CI 61.0–85.3%) for untreated and infected *T. molitor* larvae, respectively (Fig. 4d).

### Unravelling the TmDV genotype composition

**Fig. 5.**
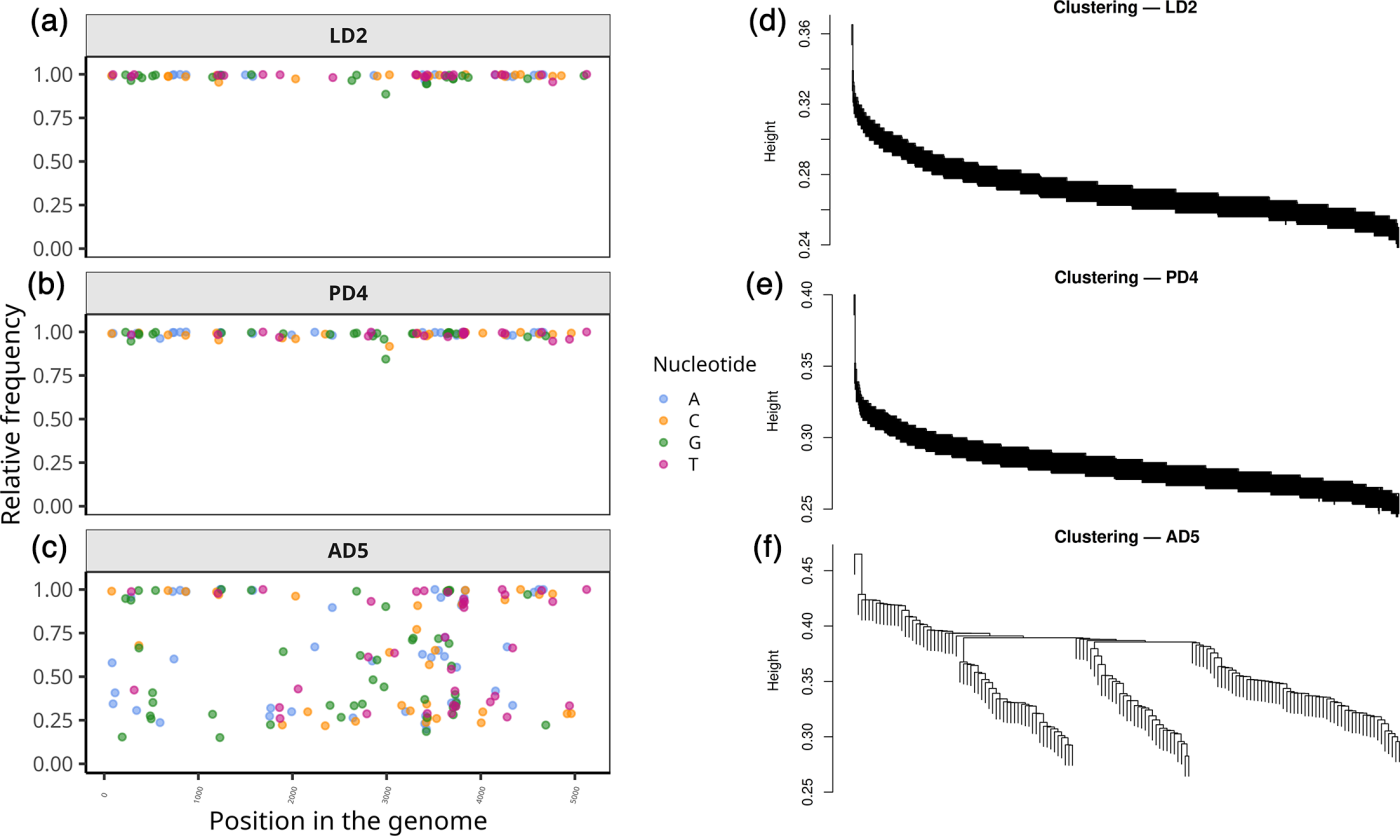
SNV frequency distribution plots (a–c) and corresponding read clustering dendrograms (d-f) for three representative samples, LD2, PD4 and AD5. Panels (a–c) show the relative frequency of each nucleotide (A, C, G, T; colour-coded), with the *x*-axis indicating the SNV positions in the genome and the *y*-axis representing relative frequency. In LD2 (a) and PD4 (b), nucleotide frequencies were close to 1 (coloured dots) or 0 (not shown), suggesting a homogeneous TmDV genotypic composition, whereas AD5 (c) represents a heterogeneous sample. Read clustering dendrograms with a long, continuous chain-like structure (d, e) confirm homogeneity of LD2 and PD4, whereas the heterogenous AD5 exhibits a more complex clustering pattern (f), indicative of three genotypes.

In addition to the determination of the TmDV consensus genome sequences from the 19 TmDV samples (Table 2), the genotypic composition of the virus in the respective samples could be further determined due to the availability of sufficiently long TmDV Nanopore reads. For this purpose, TmDV-specific reads from each sample were mapped to the reference, and all SNV positions were identified. The frequency of alternate nucleotides at these positions was then calculated and plotted to assess intra-sample genotypic variation within each sample. Based on these SNV profiles, reads of each sample were clustered by genotype using a single linkage clustering approach [10], allowing the separation of distinct viral variants based on the SNV distribution. Each cluster of reads was then extracted and used to assemble genotype-specific consensus sequences. Fig. 5 illustrates this approach by showing representative results for one sample from each developmental stage: LD2 (larvae), PD4 (pupae) and AD5 (adult). For all SNV and read clustering analyses, see (SNV frequency plots) and (linkage analysis). The SNV frequency plots display the relative frequency of the alternative nucleotides (A, T, G and C) at each detected SNV position in comparison to the reference genome of PmDV (GenBank accession number KU727766.1) (Fig. 5a–c). These plots show the relative frequency (*f*) of each of the four nucleotides (A, C, G and T) at a given variable position in the genome. A frequency of *f=*1 for one nucleotide indicates that all reads at that position carry the same nucleotide, suggesting a monomorphic site. A frequency between 0<*f*<1 for two or more nucleotides indicates the presence of multiple nucleotide types at that position, revealing sequence variation. Since LD2 and PD4 displayed relative frequencies of *f* ≈ 1, their genotypic composition was considered homogeneous, differing at the xx and yy indicated positions, respectively, from the reference genome PmDV. Their homogeneity was also reflected by the single linkage clustering of reads (Fig. 5d, e). Here, the homogeneous composition of LD2 and PD4 was reflected by the long, unbranched, chain-like dendrogram. On the contrary, AD5 exhibited a heterogeneous genotypic composition, which was indicated by numerous SNV positions with varying frequencies of 0<*f*<1 (Fig. 5c). The clustering of reads resulted in a dendrogram with three distinct branches (Fig. 5f), which proposes the presence of three TmDV genotypes within this sample. The corresponding reads were extracted from each branch of the dendrogram and assembled into individual genotypes AD5.1, AD5.2 and AD5.3 (Table 3).

**Table 3.** Summary of the 15 TmDV genotypes identified across individual samples. Genotypes were determined based on variable SNV positions and clustering of TmDV-specific Nanopore reads ranging from 2,500 to 5,500 nt in length

Sample	No. of genotype	Genotype
TmDV-LD4	2	LD4.1, LD4.2
TmDV-LH13	2	LH13.1, LH13.2, LH13.3*
TmDV-LH14	3	LH14.1, LH14.2, LH14.3
TmDV-PD1	1	PD1.1, PD1.2*
TmDV-PD2	2	PD2.1, PD2.2
TmDV-PH14	1	PH14.1, PH14.2*
TmDV-AD5	3	AD5.1, AD5.2, AD5.3
TmDV-AH14	1	AH14.1, AH14.2*, AH14.3*

*Assembly was not feasible due to low Nanopore read coverage and inadequate overlap support required for contig construction.

Among all analysed samples, all symptomatic larvae (LD1–LD5) and pupae (PD1–PD5) displayed highly homogeneous SNV patterns with relative nucleotide frequencies of *f* ≈ 1, suggesting that the assembled consensus sequences given in Table 2 accurately represented the most dominant TmDV genotype within these samples, with minor genetic variation. In contrast, nine samples, primarily from asymptomatic individuals of larvae (LH13 and LH14) and pupae (PH14), as well as from all adults – regardless of the symptom status – showed heterogeneous SNV patterns that are typical for the presence of multiple viral genotypes (). From these nine samples, single linkage clustering allowed separating the genotypes within the samples LH13, LH14, PH14, AD5 and AH14. Although some complex dendrogram structures were observed for the adult symptomatic samples (AD1–AD4), the number of TmDV reads in each branch was insufficient for genotype reconstruction. Despite their homogeneous SNV profiles, the symptomatic samples LD4, PD1 and PD2 displayed little branching in the clustering analysis, most likely reflecting a minor genotype variant within these samples. In total, 15 TmDV genotypes were reconstructed from the 19 sequenced samples. A summary of these results is provided in Table 3.

**Fig. 6.**
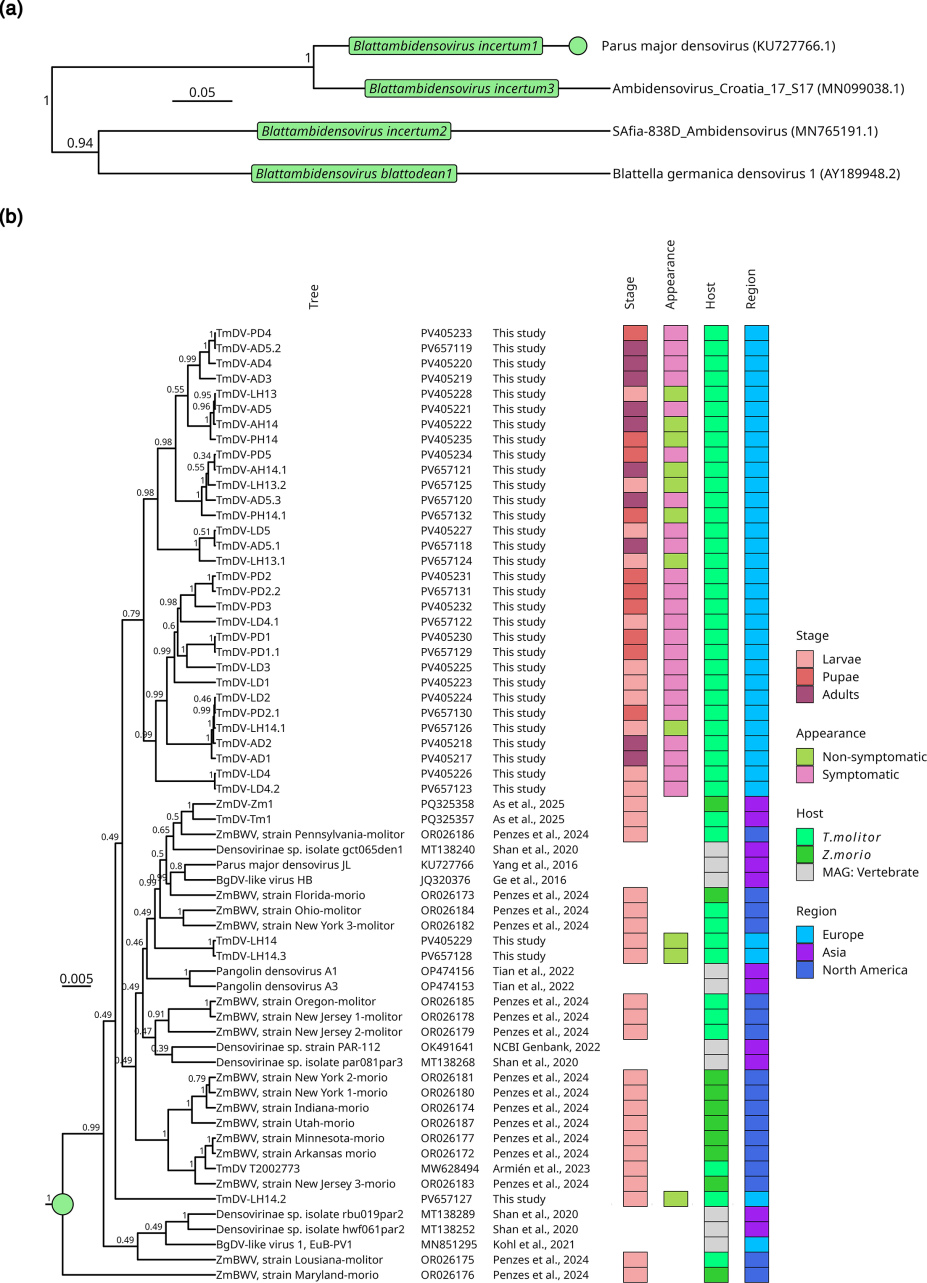
(a) Bayesian phylogeny tree showing the four representative species: *Blattambidensovirus incertum1*, *Blattambidensovirus incertum2*, *Blattambidensovirus incertum3* and *Blattambidensovirus blattodean1* from the genus *Blattambidensovirus*. Green boxes mark the species name and exemplar species are shown at the tips (from ICTV). (b) Detailed view of the species *Blattambidensovirus incertum1*, corresponding to the upper clade in (a). Posterior probabilities are displayed at the nodes. Virus names and isolate/sample identifiers are shown at the branch tips (BgDV, Blattella germanica densovirus; ZmBWV, Zophobas morio black wasting virus), followed by the NCBI GenBank accession number and corresponding publication. The heat map to the right provides metadata for each virus: Stage (life stage), appearance (symptomatic or asymptomatic), host [insect or vertebrate metagenomics analysis (MAG)] and region (geographical region of first description).

To check whether certain genotypes grouped into clades according to their host’s symptoms and life stage, all identified TmDV genotypes were phylogenetically analysed together with other known virus isolates/samples of the *Densovirinae* subfamily from the ICTV species list [37,38], including those from *Blattella germanica* [19,20], the superworm *Z. mori* [17] and from vertebrate metagenome studies [18,19] (; Fig. 6a-b). According to the Bayesian phylogenetic tree, all TmDV genotypes of this study were grouped in one common clade, except for the sequences of TmDV-LH14, -LH14.2 and -LH14.3, which were positioned in the second large clade together with densoviruses associated with *T. molitor* and *Z. morio* from America and Asia and with vertebrate studies. Examining the distribution of the TmDV specimen in more detail did not reveal any correlation between the genotypes and the life stage of the mealworm, nor with the host symptoms.

**Fig. 7.**
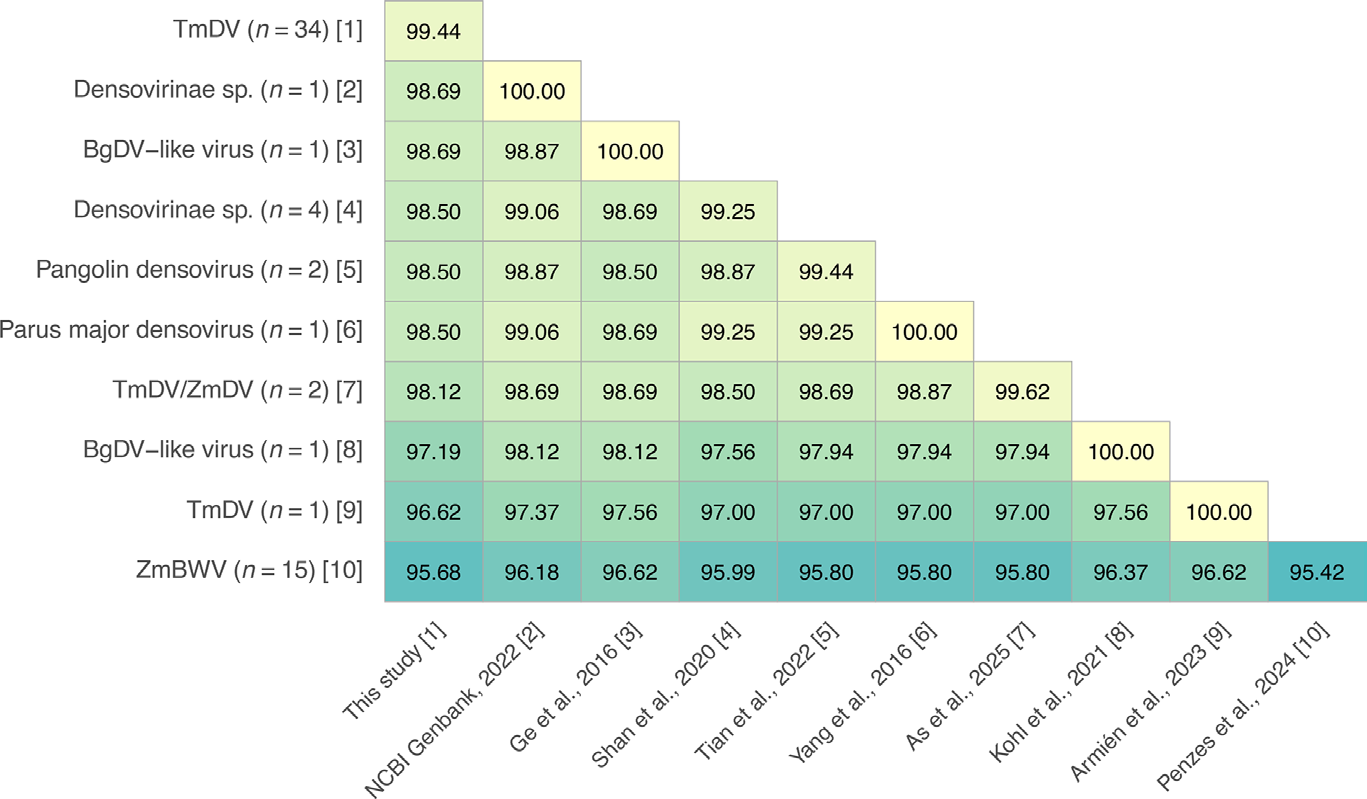
Grouped similarity heatmap of NS1 amino acid sequences from densoviruses across different studies. The numbers in each cell indicate the minimum percentage (%) of pairwise amino acid identity between the predicted NS1 proteins. The sequences of a group/dataset are numbered consecutively from [1] to [10] in square brackets. The TmDV group [1], referring to this study, includes 19 TmDV consensus sequences (Table 2) and 15 reconstructed genotypes (Table 3), adding up to 34 sequences. Rows and columns are labelled with the virus (group) names and the study or sequence references, respectively.

**Fig. 8.**
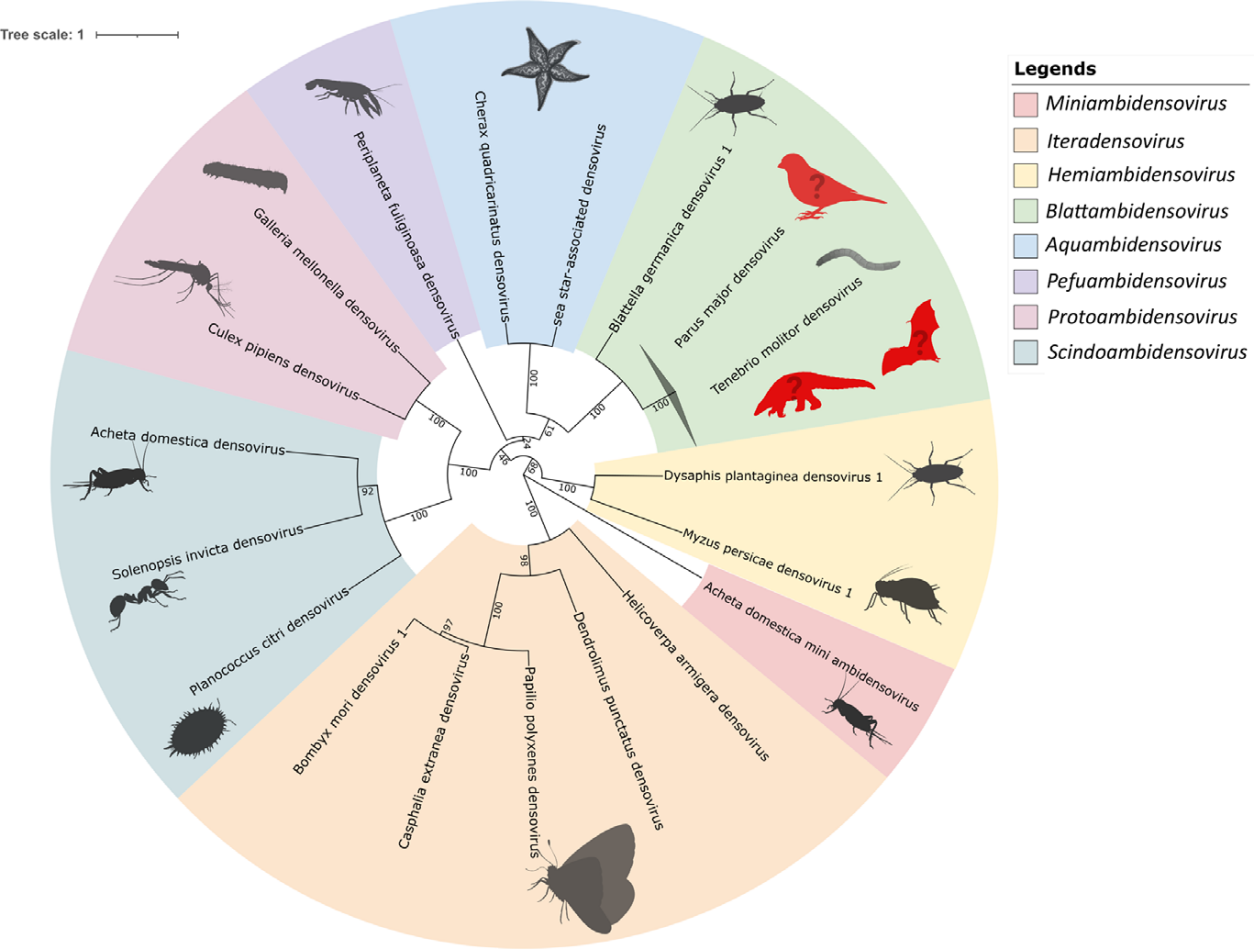
Phylogenetic overview of the subfamily *Densovirinae* (family *Parvoviridae*) and its genera [37]. The maximum likelihood tree is based on an alignment of the predicted amino acid sequences of ORF3 (NS1) of the respective taxa (, which is used for species demarcation within the family *Parvoviridae* [37,38]. The associated hosts are indicated by the animal symbols. The TmDV samples of this study fell within the genus *Blattambidensovirus* (green segment), of which vertebrate (bird, bat and pangolin) member viruses were identified by metagenomics studies with unclear host association (red animal symbols marked with a question mark). Numbers on the nodes indicate ultra-fast bootstrap (1,000 replicates) values.

To support the hypothesis that all genome sequences of Fig. 6b belong to representative viruses of the species *Blattambidensovirus incertum1*, the ICTV criterion for *Parvoviridae* species demarcation, which is an amino acid sequence similarity of >85% for the NS1 protein [37], was tested. After aligning all predicted NS1 amino acid sequences, the sequence identity was calculated and an identity matrix was created (Fig. 7). For simplification, the sequences from respective studies were grouped, and the minimum similarity value was used for each group comparison. All groups had an amino acid sequence similarity of at least 96%, fulfilling the ICTV criterion and confirming that all sequences belonged to viruses of the species *Blattambidensovirus incertum1*. To illustrate the diversity and host associations of the eight genera belonging to the *Densovirinae* subfamily [37], a phylogenetic tree based on representative NS1 amino acid sequence was constructed (Fig. 8). All TmDV sequences deciphered in this study collapsed together with TmDV and ZmBWV within the *Blattambidensovirus incertum1* clade, with PmDV as the exemplar virus. Representative host icons were symbolically added to each genus to highlight the notable discrepancy between invertebrate and putative vertebrate hosts observed within the genus *Blattambidensovirus*.

## Discussion

In this study, we confirmed TmDV as a pathogenic agent of *T. molitor* by providing clear experimental evidence of infection of TmDV and adding the mealworm to the ecological and physiological host range of the virus. Through a combination of molecular and morphological analyses, we identified TmDV across all life stages of *T. molitor*, in both symptomatic and asymptomatic individuals, suggesting potential vertical and horizontal transfer of the virus. Although viruses within the family *Densovirinae* are known to be persistent in the environment and can be transmitted mechanically between insects [12,17, 39], the detection of NS1 mRNA transcripts in larvae, pupae and adults indicates that TmDV is transcribed and replicated during host development, even in apparently healthy mealworms. This observation aligns with the findings of Penzes *et al*. [17], who found that ZmBWV remains detectable during the rearing of the superworm *Z. morio* for up to four months. In addition, our metagenomic analyses revealed a substantially lower abundance of TmDV load in the adult stage compared to larvae or pupae. Though these results are based on Nanopore read counts rather than an absolute quantification of TmDV with qPCR or similar, multiple studies from medical diagnosis of influenza virus [40,41] have demonstrated the capability of Nanopore metagenomic sequencing in providing a strong correlation between the virus amount and the proportion of viral reads in respective samples. The low abundance of TmDV in adults may be due to major physiological changes occurring during metamorphosis, including tissue remodelling, immune activation and change of life stage and appears to be typical for insect viruses exposed to a changing physiological and proteomic environment during host metamorphosis as noted for the Junonia coenia densovirus (JcDNV) and *Spodoptera frugiperda* [42], the mosquito densovirus and *Aedes aegyptii* [43] or baculoviruses [44]. Additionally, after entry into the host nucleus, the 3′ hairpin ends of the densovirus genome act as primers for the host DNA polymerase to convert the ssDNA into a dsDNA replicative form, requiring the host cell to arrest in the S-phase [45,46]. Consequently, as growth and cell division largely happen during the larval stage [47], the increased number of cells in the S-phase may result in an increased amount of the dsDNA replicative form.

Our study further revealed that the opportunistic bacterium *S. marcescens* was present in high abundance in some of the symptomatic samples. Since *S. marcescens* is not highly virulent to yellow mealworm [48], it is suggested that its proliferation may be favoured in hosts weakened by viral infection, potentially as a secondary opportunistic infection or as part of the decay process in dying insects. Interestingly, this observation is also in agreement with a finding by Dupriez *et al*. [48], who proposed *S. marcescens* as a marker for monitoring insect colony health due to its persistence in the rearing environment. In houseflies, the increase of *S. marcescens* in the gut inhibits larval growth, leading to a drop in beneficial gut bacteria and indicating immune stress [49]. Elevated levels of *Serratia* reads in diseased mealworms could serve as a warning sign of compromised colony health, although it remains unknown if the bacterium is sufficiently virulent on its own or simply capitalizes on hosts already debilitated by TmDV.

Our successful attempt to infect mealworms with purified particles of TmDV-LD2 demonstrated that TmDV is indeed pathogenic to this host. However, the severity of the disease strongly depended on the viral titre, as only mealworm larvae fed with the highest dose of 4.56×10^10^ virus particles showed clear disease symptoms of stunted growth, sluggish movement and melanization, and experienced mortality starting at 13 dpi. Although only 15 individuals were used for the re-infection studies and two larvae were scored dead, the successful establishment of a viral infection was confirmed, since the presence of TmDV prior to the infection experiment was confirmed by the absence of TmDV in the untreated negative control. In contrast, the majority of larvae exposed to lower doses remained uninfected throughout the re-infection experiment, whereas intermediate doses resulted in infected but asymptomatic larvae, suggesting that TmDV can establish a sublethal, persistent infection in *T. molitor*. This finding indicates that TmDV causes disease symptoms only above a certain infection threshold and may not uniformly kill all infected hosts. This finding contradicts previous reports of mass mortality in mealworm colonies in the USA [15,17] and Turkey [16] caused by similar densoviruses such as the ZmBWV. Additionally, abiotic factors such as stress-related conditions, which are prevalent in mass-reared insect colonies, may also influence susceptibility and disease outcome. Mass-reared insect colonies may be exposed to unfavourable conditions, e.g. overcrowding, unstable temperature or suboptimal nutrition, all known to impair immune responses and facilitate disease outbreak in insects [37,50,54]. Such environmental stressors could explain why the infection escalates into devastating outbreaks under mass-rearing conditions, whereas more controlled laboratory settings, as in our study, may lead to a persistent yet sub-lethal infection.

Genetic divergence among TmDV isolates is another plausible factor influencing their virulence. The TmDV samples detected in our study form a distinct clade, different from that of the US strains (OR026172 to OR026187), as evidenced by their phylogenetic relationship. A similar pattern has been observed with Zophobas morio densovirus, causing 90–100% mortality in a commercial superworm colony, whereas a genotype found in a healthy colony with no disease symptoms had a 99.7% nucleotide identity [55]. Likewise, the naturally attenuated strain (See Fig. 6b) ZmBWV-NJ2-molitor and a virulent ZmBWV isolate shared 97.3% genomic identity, but despite differences in virulence and capsid structure, there was no distinct phylogenetic separation between the two attenuated and virulent strains. Thus, the observed difference in virulence was not reflected in the phylogeny [17]. In the case of JcDNV, the change of just four amino acids in the capsid protein significantly reduced the virulence towards *S. frugiperda* by impairing its ability to cross the host midgut barrier Multeau *et al*. [55]. Although we succeeded in bioinformatically separating at least two TmDV genotypes from asymptomatic *T. molitor* adults using a single linkage clustering of long Nanopore reads [10,56], we could not definitely determine whether these genotypes exhibit varying virulence.

Our comprehensive phylogenetic inference and the NS1 protein distance analysis demonstrate that TmDV (this study), ZmBWV [17] and several densovirus-like sequences previously recovered from pangolin [22], bird [18,19] and bat [20,21] form a single clade and fall within a single viral species: *Blattambidensovirus incertum1*. Despite being detected in taxonomically distant organisms, their predicted NS1 amino acid sequence exhibits >96% identity, well above the ICTV species demarcation threshold of 85% [37]. Based on these findings, we propose to classify these viruses as belonging to one species, regardless of their apparent origin. However, only in the case of TmDV it has been experimentally proven – by fulfilling Koch’s third postulate – that it can infect *T. molitor*, highlighting a broader concern in modern virology, which is misassignment of host associations in metagenomics studies. Some viruses found in vertebrate viromes may not have originated from genuine infections but from physical associations. For instance, many RNA viruses detected in birds [57], bats [58,59] and other vertebrates are likely to be derived from dietary intake, gut flora or ingested arthropods, rather than representing bona fide vertebrate pathogens. Similarly, Dennis *et al*. [60] provided evidence that the circovirus (family *Citroviridae*) sequences detected in vertebrates were more plausibly of invertebrate origin. These findings challenge the assumption that metagenomics detection equates to host infection. As emphasized by Chang *et al*. [61] and [62], host–virus attribution without experimental evidence is a major pitfall of metagenomic studies. Since its initial identification [15], *Blattambidensovirus incertum1* has been associated with a diverse variety of hosts, including both invertebrates and vertebrates, which led to uncertainty about its true host origin. While Penzes *et al*. [17] demonstrated experimental infection of *Z. morio* with ZmBWV, our study provides further evidence by demonstrating replication of TmDV in *T. molitor*. Together, these findings establish a clear link between the virus and members of the Tenebrionidae family (Coleoptera). In light of this expanded and experimentally validated host association, the arthropod association of all other members of the *Densovirinae* and the lack of any evidence for vertebrate infection, we hypothesize that true hosts of *Blattambidensovirus incertum1* viruses are insects and possibly other invertebrates.

## Supplementary material

10.1099/jgv.0.002211 Supplementary Material 1.

## Abbreviations

ICTV: International Committee on Taxonomy of Viruses
JcDNV: Junonia coenia densovirus
MAGs: metagenome-assembled genomes
NCBI: National Center for Biotechnology Information
NS: non-structural
ORF: open reading frame
PmDV: Parus major densovirus
qPCR: quantitative PCR
SNVs: single-nucleotide variants
TmDV: Tenebrio molitor densovirus
