# Supplementary material for: No longer uncertain: the validation of tenebrionid insects as hosts of Blattambidensovirus incertum1 isolates by phylogeny and infection studies
Source: J Gen Virol. 2026 Feb 10;107(2):002211. doi: 10.1099/jgv.0.002211 (PMC12890311; doi:10.1099/jgv.0.002211)
Supplement: Supplementary Material 1. [file jgv-107-02211-s001.pdf]

Table S1. Virus sequences of *Densoviriniae* used for phylogenetic inference and their respective GenBank accession number.

Virus	Accession Number
Blattella germanica densovirus-like virus HB	JQ320376
Parus major densovirus JL	KU727766
Blatella germanica densoviruses-like virus 1 isolate EuB-PV1	MN851295
Densovirinae sp. isolate gct065den1	MT138240
Densovirinae sp. isolate hwf061par2	MT138252
Densovirinae sp. isolate par081par3	MT138268
Densovirinae sp. isolate rbu019par2	MT138289
Tenebrio molitor densovirus T2002773	MW628494
Densovirinae sp. strain PAR-112	OK491641
Pangolin densovirus A3	OP474153
Pangolin densovirus A1	OP474156
Zophobas morio black wasting virus, strain Arkansas morio	OR026172
Zophobas morio black wasting virus, strain Florida-morio	OR026173
Zophobas morio black wasting virus, strain Indiana-morio	OR026174
Zophobas morio black wasting virus, strain Lousiana-molitor	OR026175
Zophobas morio black wasting virus, strain Maryland-morio	OR026176
Zophobas morio black wasting virus, strain Minnesota-morio	OR026177
Zophobas morio black wasting virus, strain New Jersey 1-molitor	OR026178
Zophobas morio black wasting virus, strain New Jersey 2-molitor	OR026179
Zophobas morio black wasting virus, strain New York 1-morio	OR026180
Zophobas morio black wasting virus, strain New York 2-morio	OR026181
Zophobas morio black wasting virus, strain New York 3-molitor	OR026182
Zophobas morio black wasting virus, strain New Jersey 3-morio	OR026183
Zophobas morio black wasting virus, strain Ohio-molitor	OR026184
Zophobas morio black wasting virus, strain Oregon-molitor	OR026185
Zophobas morio black wasting virus, strain Pennsylvania-molitor	OR026186
Zophobas morio black wasting virus, strain Utah-morio	OR026187
Tenebrio molitor densovirus PH14.1	PV657132
Tenebrio molitor densovirus Tm1	PQ325357
Zophobas morio densovirus Zm1	PQ325358
Tenebrio molitor densovirus AD1	PV405217
Tenebrio molitor densovirus AD2	PV405218

Tenebrio molitor densovirus AD3	PV405219
Tenebrio molitor densovirus AD4	PV405220
Tenebrio molitor densovirus AD5	PV405221
Tenebrio molitor densovirus AD5.1	PV657118
Tenebrio molitor densovirus AD5.2	PV657119
Tenebrio molitor densovirus AD5.3	PV657120
Tenebrio molitor densovirus AH14	PV405222
Tenebrio molitor densovirus AH14.1	PV657121
Tenebrio molitor densovirus LD1	PV405223
Tenebrio molitor densovirus LD2	PV405224
Tenebrio molitor densovirus LD3	PV405225
Tenebrio molitor densovirus LD4	PV405226
Tenebrio molitor densovirus LD4.1	PV657122
Tenebrio molitor densovirus LD4.2	PV657123
Tenebrio molitor densovirus LD5	PV405227
Tenebrio molitor densovirus LH13	PV405228
Tenebrio molitor densovirus LH13.1	PV657124
Tenebrio molitor densovirus LH13.2	PV657125
Tenebrio molitor densovirus LH14	PV405229
Tenebrio molitor densovirus LH14.1	PV657126
Tenebrio molitor densovirus LH14.2	PV657127
Tenebrio molitor densovirus LH14.3	PV657128
Tenebrio molitor densovirus PD1	PV405230
Tenebrio molitor densovirus PD2	PV405231
Tenebrio molitor densovirus PD3	PV405232
Tenebrio molitor densovirus PD4	PV405233
Tenebrio molitor densovirus PD5	PV405234
Tenebrio molitor densovirus PH14	PV405235
Tenebrio molitor densovirus PD2.2	PV657131
Tenebrio molitor densovirus PD2.1	PV657130
Tenebrio molitor densovirus PD1.1	PV657129
Solenopsis invicta densovirus	KC991097
Acheta domestica densovirus	HQ827781
Planococcus citri densovirus	AY032882
Culex pipiens densovirus	AY032882
Galleria mellonella densovirus	L32896
Myzus persicae densovirus 1	AY148187
Dysaphis plantaginea densovirus1	FJ040397
Sea star-associated densovirus	KM052275
Cherax quadricarinatus densovirus	KP410261
Periplaneta fuliginoasa densovirus	AF192260
Blatella germanica densovirus 1	AY189948
Acheta domestica mini ambidensovirus	KF275669
Helicoverpa armigera densovirus	HQ613271
Casphalia extranea densovirus	AF375296
Bombyx mori densovirus 1	AY033435
Papilio polyxenes densovirus	JX110122
Dendrolimux punctatus densovirus	AY665654
Penaeus stylirostris penstyldensovrius	AF273215
Fenneropenaeus chinesens hepandensovirus	ADK63426

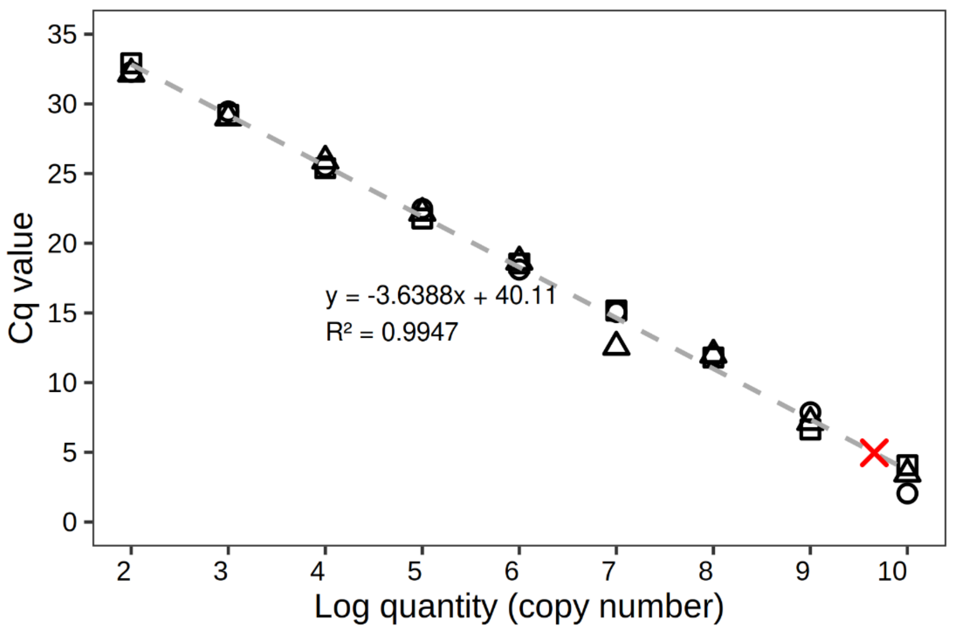

Figure S1: qPCR standard curve for TmDV quantification. The standard curve was based on plasmid pEX-A128::TmDV-NS1(89bp), which contained a copy of an 89 bp fragment of ORF NS1 from TmDV. Since plasmid length and concentration were precisely known, it was used to create an absolute standard curve to approximate the viral genome copy number in the TmDV-LD2 stock suspension. The symbols represent the three replicates (dilution series) of the standard curve. The red X marks the measurement of the TmDV-LD2 sample.

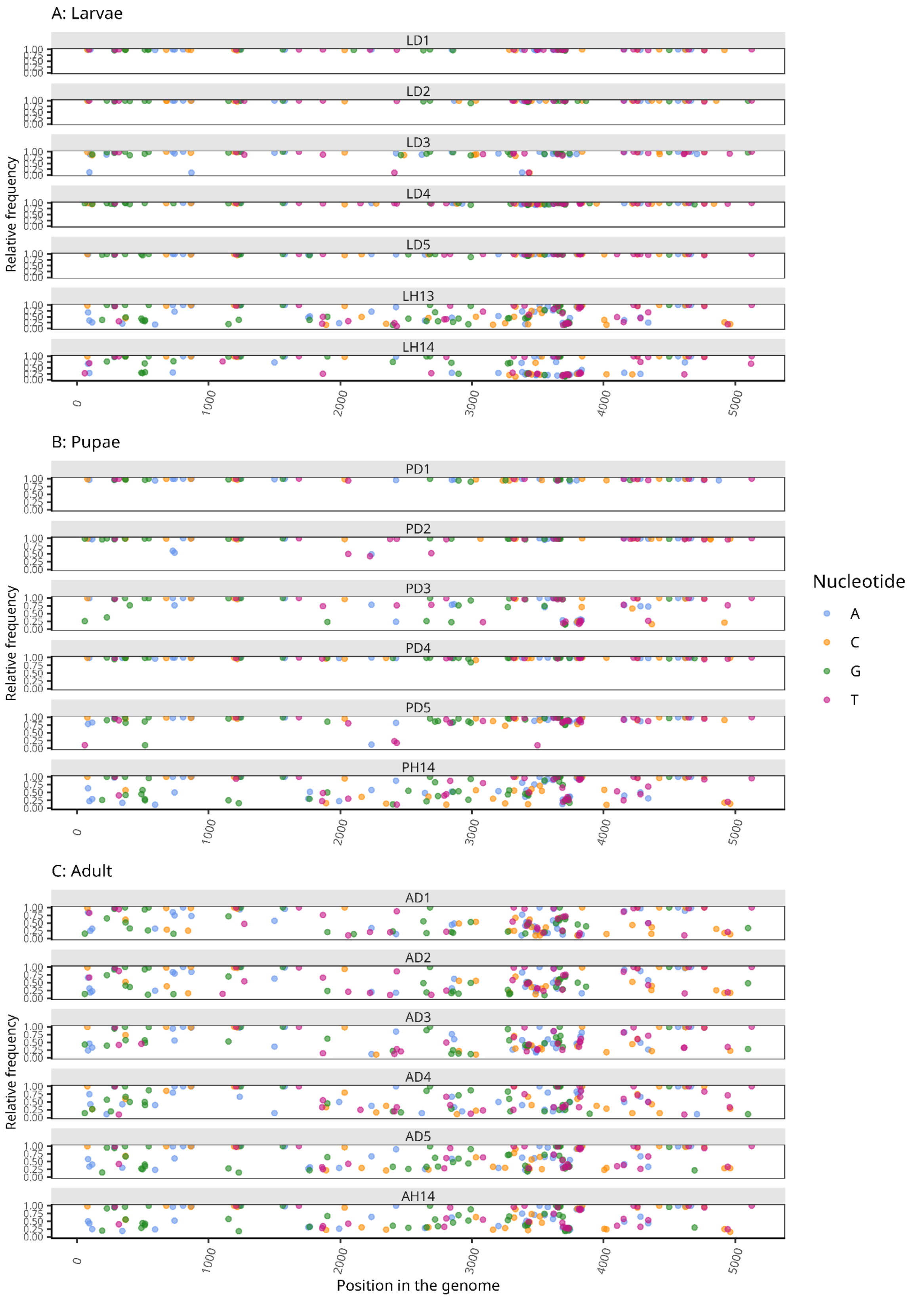

Figure S2: Nucleotide frequency plots of identified single nucleotide variant (SNV) positions of TmDV across all *Tenebrio molitor* samples from three developmental stages: (A) Larvae, (B) Pupae and (C) Adults.

Each point represent the relative frequency of one of the four nucleotide (with different colors) observed at a given SNV position within a sample. Frequencies were calculated by mapping TmDV reads to the exemplar species of *Blattambidensovirus incertum1* (KU727766.1). A frequency of f = 1 at a given nucleotide position indicates that all reads at that position carry the same nucleotide different from the reference genome, while 0 < f < 1 for multiple nucleotides suggest sequence variation. Positions with f = 0 (= identity with reference) are not indicated.

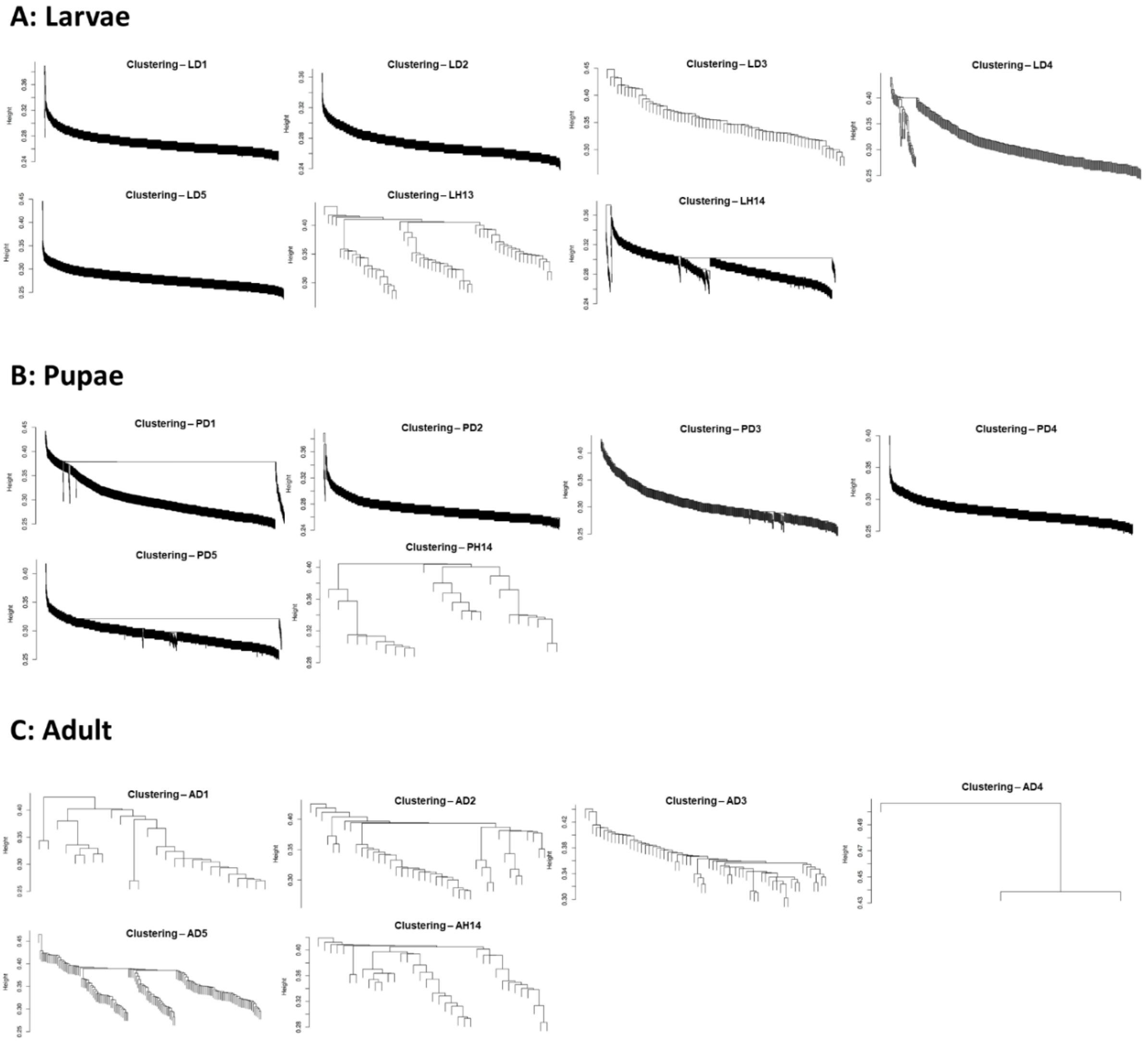

Figure S3: Single-linkage clustering of reads across all samples from three developmental stages: (A) Larvae, (B) Pupae, and (C) Adults, using sing nucleotide variant (SNV) positions as an anchor for the analysis. Chain-like, unbranched dendrograms (LD1, LD2, LD3, LD5, PD3, PD4) indicate a homogenous viral population with minimal SNV co-segregations. In contrast, samples with heterogeneous patterns (LD4, LH13, LH14, PH14, AD1, AD2, AD3, AD4, AD5, AH14) displayed branched dendrograms, suggesting the presence of multiple TmDV genotypes within the same host. Minor branches, indicating limited variation were observed in samples PD1, PD2 and PD5. In these cases, reads were extracted and genotype reconstruction was attempted, whenever it was possible.
